# Level of food consumption score and associated factors among households in Konso Zone, Southwestern Ethiopia: a community-based cross-sectional study

**DOI:** 10.3389/fnut.2024.1481458

**Published:** 2024-10-28

**Authors:** Kasata Markos, Samson Kastro Dake, Fithamlak Solomon Bisetegn, Debritu Nane

**Affiliations:** ^1^Arbaminch College of Health Science, Arbaminch, Ethiopia; ^2^Department of Reproductive Health and Nutrition, School of Public Health, College of Health Science and Medicine, Wolaita Sodo University, Wolaita Sodo, Ethiopia

**Keywords:** food consumption score, factors, households, dietary diversity, Konso

## Abstract

**Background:**

Despite several attempts made in the developing world to improve overall food consumption patterns, it is still a major problem. However, there is limited literature on evidence of FCS, particularly in the study area.

**Objective:**

This study aimed to assess the level of food consumption score and associated factors among households in Konso Zone, Southwestern Ethiopia.

**Methods:**

A community-based cross-sectional study was conducted among 488 households in Konso Zone, southern Ethiopia. Data were collected using an interviewer-administered, pre-tested, structured questionnaire. We assessed FCS through a seven-day dietary recall of food consumption. The households were labeled as “poor FCS” when they had a food composite score of <21.5; “borderline FCS” when they had a food composite score of 21.5–35; and “acceptable FCS” when they had a food composite score of >35 during the reference period. The data were entered into Epi-Data version 3.1 and exported to Statistical Package for Social Science (SPSS) version 25 for analysis. The *p*-value, adjusted odds ratios (AORs), and 95% confidence intervals (CIs) were used to identify the associated factors.

**Results:**

The acceptable FCS among the study participants was 68.3% (95% CI: 63.9, 72.4), whereas 17.4% (95% CI: 14.1, 21.2) were borderline and 14.3% (95% CI: 11.3, 17.8) were poor. There was a higher level of acceptable FCS among households with household heads who were married (AOR = 2.22; 95% CI: 1.08, 4.58), aged 18–24 years (AOR = 0.13, 95% CI: 0.05, 0.30), farmers (AOR = 0.22, 95% CI: 0.13, 0.39), and attended formal education (AOR = 2.68, 95% CI: 1.65, 4.21).

**Conclusion:**

The prevalence of acceptable FCS was found to be low. The age of the household head, marital status, occupation, and residence had a significant relationship with the study outcome. Therefore, interventions should target younger-headed and unmarried households. Policies and programs should support the ownership of farmland and promote formal education.

## Introduction

Access to a safe, adequate, and balanced diet is a basic human right (1). Unfortunately, this basic right is denied in many low and middle-income countries (LMICs) (2). Consequently, all life stages are vulnerable to malnutrition. An inadequate intake of micronutrients, an inability to obtain nutrient-rich foods, a lack of diversified foods, or infrequent dietary consumption may result in one or more forms of malnutrition (3, 4).

Food security is a critical aspect of public health, encompassing access to sufficient, safe, and nutritious food necessary for an active and healthy life (5, 6). One widely utilized measure of food security is the Food Consumption Score (FCS), which assesses the dietary diversity, food frequency, and the relative nutritional importance of different food groups based on a seven-day recall of food consumed at the household level. Additionally, FSC provides a clue about the usual dietary pattern as it includes a 7-day dietary recall period. It is a composite proxy indicator of food quality (5). As an indicator of food security, the FCS reflects the variety of foods consumed over a specific period, which is vital for ensuring adequate intake of essential nutrients. Research has demonstrated a strong correlation between higher FCS values and improved nutritional outcomes, highlighting its importance in identifying households at risk of malnutrition (5).

FCS enables the categorization of households into different levels of food insecurity; secure, moderately insecure, and severely insecure facilitating targeted interventions and resource allocation (7). FCS < 35 may indicate household food insecurity (6). The prevalence of households with acceptable FCS provides information on the current dietary intake of people. It helps in deciding the most appropriate type and scale of interventions. It also enhances inputs into nutrition program design and improves the measurement of the impact of food-based interventions (8).

Most of the world’s hungry people live in developing countries, where 12.9% of the population is undernourished. The highest prevalence of hunger occurred in Sub-Saharan Africa (SSA) countries, with one in four people undernourished (9, 10). SSA has hardly confronted the food crisis and unacceptable household FCS because of food insecurity (11).

The food security scenario in Ethiopia was dramatically decreasing, with the number of food-insecure people increasing from time to time (12). In the past two decades, Ethiopia has made significant progress in development by minimizing poverty and increasing investments in basic social services. Despite several attempts made in Ethiopia to improve the overall food consumption pattern, it still remains a major problem (13).

Studies conducted in Ethiopia have shown that food consumption is not sufficient to meet the nutrient demands of people (6, 14). In 2011, the World Food Program (WFP) reported that the acceptable national FCS coverage in Ethiopia was 74% (6). A facility-based study conducted in 2018 stated that the magnitude of acceptable FCS among pregnant women was 81.5% (14). Another community-based study among pregnant women reported that the prevalence of acceptable FCS was found to be 54.5% (15).

Unacceptable FCS due to food insecurity is currently a critical public health and social issue that requires immediate attention from policymakers and other decision-makers (16–18). Unacceptable food consumption patterns are a persistent issue that varies in degree and have a negative impact on individuals, households, and social classes as well. For this reason, it is important to understand how food consumption patterns occur across different socio-economic and socio-demographic characteristics to meet specific needs by implementing appropriate policies, programs, and other nutritional intervention initiatives (19, 20).

Assessing FCS provides insights into dietary diversity and the nutritional quality of food consumed, which is essential for identifying malnutrition and micronutrient deficiencies (21) Understanding the factors associated with food consumption enhances the community’s resilience against shocks like economic downturns or climate change, allowing households to maintain stable food access (22). Even though there have been some studies conducted to assess the prevalence of FCS among pregnant women in Ethiopia, there is a paucity of evidence on the prevalence of acceptable FCS at the household level in Ethiopia and other parts of the world. Thus, this study assessed the level of FCS and associated factors among households in Konso Zone, Southwestern Ethiopia.

## Materials and methods

### Study setting and design

A community-based cross-sectional study was conducted from November 01 to 15, 2021 in Konso Zone. Konso Zone is found in Southwestern Ethiopia. The zone has three Woredas (Kena, Segen zuria and Karat zuria), Kolimme Cluster, and Karat Town Administration. Karat is the administrative town of the Zone, located around 554 kilometeres from the capital city of Ethiopia, Addis Ababa. The zone has a total population of 323,426, of whom 51% (164,947) are females and 49% (158,479) are males. It has 66,005 estimated households (23). In the zone, there are fourteen health centers and two primary hospitals. The Konso economy is based on intensive agriculture using terracing and irrigation on mountain slopes. In addition, agriculture is the major means of livelihood and potential income generating source for the people in Konso. Its agro-ecology is mostly Woyna-Dega (23).

### Population and sampling

All households in Konso Zone were the source population. Households in the selected administrative centers of the zone were the study population. The study units were randomly selected households of the study *kebeles* (small administrative units). Households that lived for at least 6 months in the study *kebeles* were included in the study. We interviewed participants aged above 18 years Individuals who are mentally ill and/or physically incapable of communicating and listening were excluded.

The sample size was calculated by using a single-population proportion formula; the proportion (p) of acceptable FCS taken from the national FCS coverage in Ethiopia was 74% (6), 95% level of confidence with a 5% margin of error (d = 0.05), a design effect of 1.5, and a 10% non-response rate were considered. Finally, the sample size was **488.**

There are 53 *kebeles* (lower administrative units) in the study area. First, 15 *kebeles* were selected by using the simple random sampling (SRS) technique. Then, the total sample size was allocated to the selected *kebeles* proportional to the number of households in each *kebele*. By using the list of eligible households in each *kebele* as a sampling frame, study households were selected using SRS. Finally, we selected the person who was mainly responsible for the purchase and preparation of food in the household for the interview.

### Data collection

Data was collected using an interviewer-administered structured questionnaire. The data collection team consisted of five data collectors (diploma health professionals) and two supervisors (BSc, health professionals). The questionnaire was developed based on the standardized WFP eight food frequency questions, the Ethiopian Demographic and Health Survey, and different literature (5, 14, 15, 24). It contained socio-demographic characteristics, household assets, household food taboos, and food consumption questions. It was prepared in English and translated into local language (*konsogna*), and again retranslated back to English to maintain consistency.

The local food items were categorized into eight food groups by using the Ethiopian food composition table and its scientific food group (25). All consumption frequencies of foods in the same group were summed, and when the value of each group result was above 7, it was recoded as 7. Then the value was multiplied by its assigned weight. Finally, the weights of food group’s scores were summed to obtain the FCS to determine the status of households (5) (Table 1).

**Table 1:** World food program eight food groups with each standard weight, 2015.

Food groups	WFP standard name	Weight
Cereals, tubers and roots	Main staples	2
Pulses	Pulses	3
Milk and dairy	Dairy	4
Meat, fish and eggs	Meat/fish/eggs	4
Vegetables	Vegetables	1
Fruits	Fruits	1
Oil and fats	Fats	0.5
Sugar	Sugar	0.5
Spices	Condiments	0

This table categorizes local food items into eight food groups using the Ethiopian Food Composition Table and its scientific food group classifications. The table also presents the standard weights assigned to each group, as defined by the WFP. For each household, the consumption frequencies of foods within the same group were summed, and if the total frequency exceeded 7, it was recoded as 7. The recoded values were then multiplied by the assigned weight for each food group. The final FCS was calculated by summing the weighted scores of all food groups to determine the food security status of households. WFP, world food program.

### Variables of the study

#### Dependent variable

**FCS:** measures dietary diversity, food frequency, and the relative nutritional importance of food groups based on a 7 day recall of food consumed at the household level. It was calculated as follows: FCS = W_staple_F_staple_ + W_pulse_F_pulse_ + W_veg_F_veg_ + W_fruit_F_fruit_ + W_animal_F_animal_ + W_sugar_ F_sugar_ + W_dairy_F_dairy_ + W_oil_F_oil,_ where F_i_ is the frequency of food consumption (number of days for which each food group was consumed during the past 7 days) and W_i_ is the weight of each food group (5).

The categories of the FCS are defined as follows: a Poor FCS ranges from 0 to 21; a Borderline FCS falls between 21.5 and 35; and an Acceptable FCS exceeds 35 (5).

#### Independent variables

The independent variables consist of various socio-demographic and economic factors, including residence, income, family size, age, sex, religion, occupation, educational status, marital status, household heads, and wealth index (Table 2).

**Table 2:** Summary of independent variables.

Variable	Type	Categories/description
Residence	Categorical	Urban, rural
Income	Continuous	Monthly income in Ethiopian Birr
Family size	Count	Total number of members in the household
Age	Continuous	Age of the respondent in years
Sex	Categorical	Male, female
Religion	Categorical	Protestant, Orthodox, Muslim, Traditional
Occupation	Categorical	Government employee, merchant, farmer, house wife, Daily Laborer, Student
Educational status	Categorical	No formal education, primary education, secondary education, college and above
Marital status	Categorical	Currently married, not married
Household heads	Categorical	Primary decision-maker in the household
Wealth Index	Categorical	Poorest, poorer, middle, richer, richest (derived from PCA analysis)

Overview of socio-demographic and economic variables, including residence, income, family size, age, sex, religion, occupation, educational status, marital status, household heads, and wealth index.

Wealth index: The composite indicator of socioeconomic status and household amenities computed by the application of principal component analysis (PCA). Then, the wealth index was converted into quartiles and ranked as poorest, poorer, middle, richer, and richest (26).

### Data management and analysis

A pilot study was conducted among 5% of respondents who were not included in the study samples, and the necessary amendments were done. Two days of training was provided for the data collectors and supervisors. The supervisors checked questionnaires for completeness on a daily basis. We assured confidentiality to minimize social desirability bias.

Data entry was done using Epi-Data Version 3.1 and cleaned, and then the data set was exported to the Statistical Package for Social Science (SPSS) version 25. An ordinal logistic regression analysis model was carried out using this software. The dependent variable was categorized as poor (FCS = 0–21), borderline (FCS = 21.5–35) and acceptable (FCS > 35). Variables with a variance inflation factor < 5, tolerance value >0.2, and/or Pearson correlation coefficient < 0.5 were used to assure no multi-collinearity among predictor variables.

Model fitting information and tests of model effects were used to prove whether there is a significant relationship between dependent and independent variables (−2Log Likelihood = 530.00, χ2 = 272.61, *p* < 0.001) and significant contribution of the predictor variables in the final model, respectively. *p* < 0.05 declared statistical significance. Pearson and Deviance statistic test was checked to declare the model is fit; *p*-value >0.05 declared the model is fit. The Pseudo R-square tolled the model accounts from 34.0 to 53.5 percent of variance. The test of Parallel Lines assured that the location parameters are different across response categories, *p* = 0.176; this *p* > 0.05 declare the model is fit. Statistical significance level, adjusted odds ratios (AORs) with their 95% confidence intervals (CI) were used to identify factors associated with the study outcome. Finally, results were presented using numerical summary measures, tables, and figures.

## Results

### Socio-demographic characteristics

Of the 488 selected households, 476 were included in the study, resulting in a response rate of 97.5%. The mean age of study participants was 33.97 years, and the mean age of household heads was 38.08 years. The majority of participants were married, female, Konso by their ethnicity, Protestant religion followers, had no formal education, and farmers by their occupation. Similarly, most household heads were married, male, Konso by their ethnicity, Protestant by their religion, and farmers by their occupation, with nearly half having no formal education. Detailed Socio-Demographic information found in the Tables 3, 4.

**Table 3:** Socio-demographic characteristics of respondents in Konso Zone, Southwestern Ethiopia, 2021 (*n* = 476).

Variables	Category	No. (%)
Age of respondent (year)	18–24	75 (15.8)
	25–34	194 (40.8)
	35–49	169 (35.5)
	≥50	38 (8.0)
Marital status of respondent	Currently married	446 (93.7)
	Currently not married^**a**^	30 (6.3)
Gender of respondent	Male	20 (4.2)
	Female	456 (95.8)
Religion of respondent	Protestant	336 (70.6)
	Orthodox	113 (23.7)
	Muslim	4 (0.8)
	Traditional	23 (4.8)
Ethnicity of respondent	Konso	445 (93.5)
	Others^**b**^	31 (6.5)
Educational status of respondent	No formal education	306 (64.3)
	Primary education	93 (19.5)
	Secondary education	32 (6.7)
	College and above	45 (9.5)
Occupation of respondent	Gov’t employee	36 (7.6)
	Merchant	44 (9.2)
	Farmer	293 (61.6)
	House wife	56 (11.8)
	Daily laborer	22 (4.6)
	Student	25 (5.3)

This table presents the socio-demographic and economic characteristics of 476 respondents in Konso Zone, Southwestern Ethiopia. The characteristics include variables such as age, marital status, gender, religion, ethnicity, education level, and occupation. a(*n*) = single (12), divorced (4), and widowed (14); b(*n*) = Derashe (5), Amaro (2), Ali (2), Burji (3), Oromo (4), Amhara (8), Tigray (2), Wolayta (3), and Gamo (2).

**Table 4:** Socio-demographic and economic characteristics of households and household heads of Konso Zone, Southwestern Ethiopia, 2021 (*n* = 476).

Variables	Category	No. (%)
Age	18–24	26 (5.5)
	25–34	161 (33.8)
	35–49	223 (46.8)
	≥50	66 (13.9)
Gender of HH head	Male	434 (91.2)
	Female	42 (8.8)
Religion of HH head	Protestant	310 (65.1)
	Orthodox	109 (22.9)
	Muslim	4 (0.8)
	Traditional	53 (11.1)
Ethnicity of HH head	Konso	456 (95.8)
	Others^a^	20 (4.8)
Educational status of HH head	No formal education	228 (47.9)
	Primary education	114 (23.9)
	Secondary education	26 (5.5)
	College and above	108 (22.7)
Occupation of household head	Gov’t employee	91 (19.1)
	Merchant	54 (11.3)
	Farmer	267 (56.1)
	Daily laborer	41 (8.6)
	Others^b^	23 (4.8)
Marital status of HH head	Currently married	447 (93.9)
	Currently not married	29 (6.1)
HHs’ residence	Urban	104 (21.8)
	Rural	372 (78.2)
Households’ family size	≤2	27 (5.7)
	3–5	159 (33.4)
	≥6	290 (60.9)
Wealth index	Poorest	95 (20.0)
	Poorer	95 (20.0)
	Middle	96 (20.2)
	Richer	96 (20.2)
	Richest	94 (19.7)

This table presents the socio-demographic and economic characteristics of 476 households and their heads in Konso Zone, Southwestern Ethiopia. The characteristics include variables such as age, gender, religion, ethnicity, education level, marital status, education level, household size, and wealth index indicators. a(*n*) = Derashe (1), Amaro (1), Ali (2), Burji (3), Oromo (2), Amhara (3), Tigray (3), and Wolaita (4); b(*n*) = non-governmental employee (10), housewife (9), and student (4); and c(*n*) = divorced (6) and widowed (23). HH, household.

Out of 476 interviewed households, majority were rural residents and the most frequent family size was more than five. The mean of households’ estimated average monthly income was 3856.13 Ethiopian birr (Table 4).

### Food consumption score

**Figure 1:**
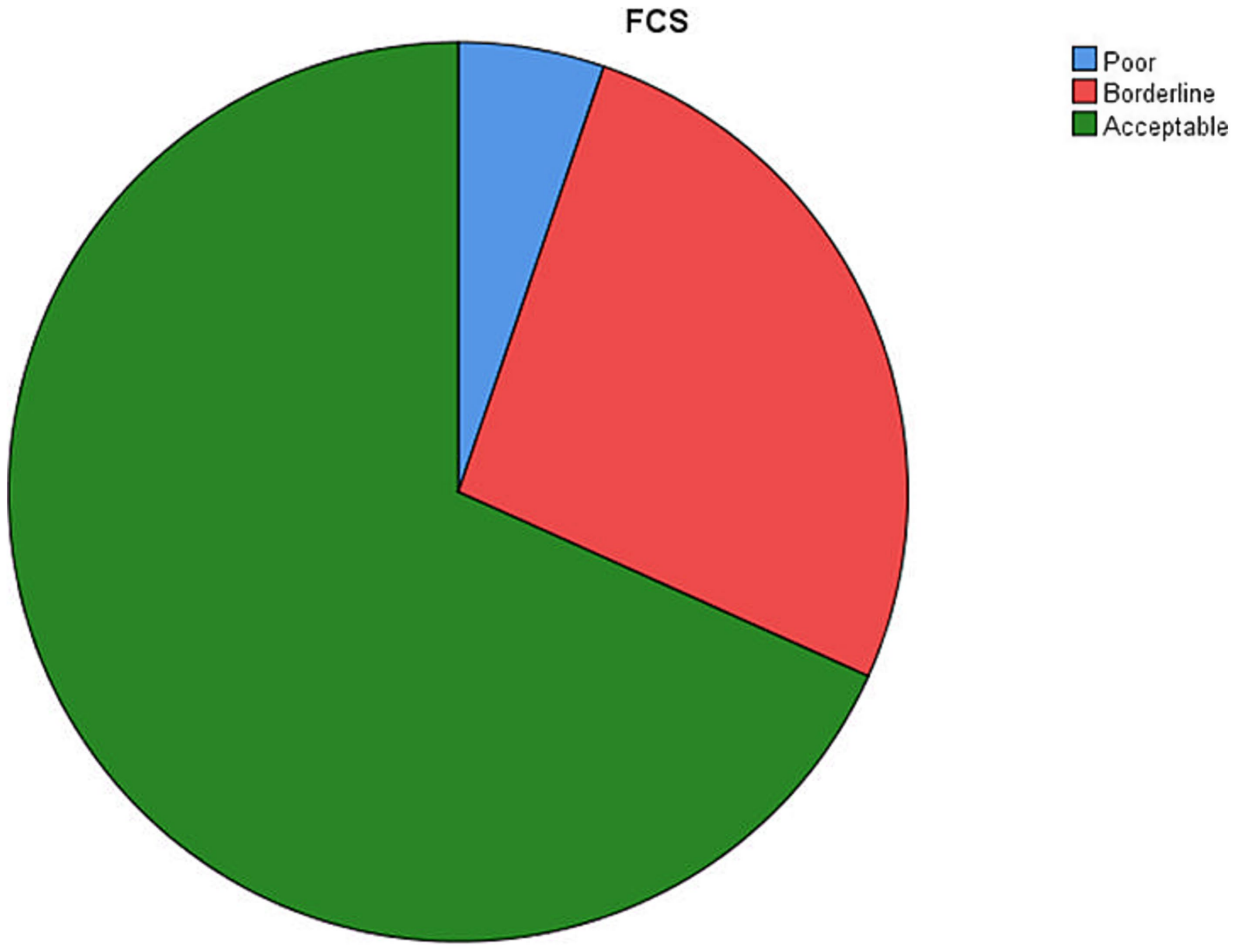
The prevalence of FCS in Konso Zone, Southwestern Ethiopia, 2021. Participants with poor FCS were 25 (5.2%), borderline FCS were 126 (26.5%), and acceptable FCS were 325 (68.3%).

In this study, it was found that more than two third, 325 (68.3%: 95% CI: 63.9, 72.4) of the study participants had acceptable FCS (Figure 1).

### Factors associated with level of FCS

The tests of model effects under ordinal logistic regression proved that age of the household head, marital status of the household head, occupation of the household head, and educational status of the household head had a significant association with FCS (*p* < 0.05).

The odds of falling on acceptable FCS were 87% lower (AOR = 0.13, 95% CI: 0.05, 0.30) for households whose household heads are aged 18–24 compared to those aged 30 years and above. Similarly, the odds of falling on acceptable FCS were 78% lower (AOR = 0.22, 95% CI: 0.13, 0.39) for households whose household heads are aged 25–29 years compared to those aged 30 years and above. In addition, households with married household heads had a 2.22 (AOR = 2.22, 95% CI: 1.08, 4.58) times higher chance of having acceptable FCS compared to their counterparts. Regarding the occupational status, households with farmer household heads had a 4.7 (AOR = 4.70, 95% CI: 2.75, 8.03) times higher chance of having an acceptable FCS compared to the other occupational statuses. Households with household heads who have attended formal education had a 2.68 (AOR = 2.68, 95% CI: 1.65, 4.21) times higher odds of having acceptable FCS compared to households with household heads who did not attend formal education (Table 5).

**Table 5:** Factors associated with level of FCS among households in Konso Zone, Southwestern Ethiopia, 2021 (*n* = 476).

Variables	Food consumption score [no (%)]			COR (95% CI)	AOR (95% CI)
	Poor	Borderline	Acceptable		
**Age of HH head**					
18–24	8 (32.0)	8 (6.3)	10 (3.1)	0.15 (0.64, 0.33)	0.13 (0.05, 0.30)**
25–29	9 (36.0)	27 (21.4)	41 (12.6)	0.38 (0.23, 0.63)	0.22 (0.13, 0.39)**
30+	8 (32.0)	91 (72.2)	274 (84.3)	1	1
**Marital status of HH head**					
Currently married	60 (88.2)	77 (92.8)	310 (95.4)	3.43 (0.9, 13.04)	2.22 (1.08, 4.58)*
Currently not married^a^	8 (11.8)	6 (7.2)	15 (4.6)	1	1
**Occupation of HH head**					
Farmer	18 (72.0)	105 (83.3)	144 (44.3)	5.28 (3.32, 8.38)	4.70 (2.75, 8.03)**
Others^b^	7 (28.0)	21 (16.7)	181 (55.7)	1	1
**Educational status of HH head**					
Formal education	20 (80.8)	85 (67.5)	122 (37.5)	4.01 (2.65, 6.05)	2.68 (1.65, 4.21)**
No formal education	5 (19.2)	41 (32.5)	203 (62.5)	1	1

This table outlines the factors associated with varying levels of Food Consumption Score (FCS) among 476 households in Konso Zone, Southwestern Ethiopia. The table includes socio-demographic variables that were analyzed to determine their influence on FCS levels, indicating the food consumption score of the households. a(*n*) = divorced (6) and widowed (23); b(*n*) = non-governmental employee (10), housewife (9), and student (4). * Statistically significant at *p* < 0.05, ** statistically significant at *p* < 0.001. HH, household; COR, crude odds ratio; AOR, adjusted odds ratio.

## Discussion

Unacceptable (borderline and poor) FCS is a major public health problem that needs strengthening of nutrition interventions (27, 28). Therefore, this study assessed the level of FCS and associated factors among households in Konso Zone. In this study, the proportions of poor, borderline, and acceptable FCS were 5.2, 26.5, and 68.3%, respectively. Age, marital status, occupation, and educational status of the household heads had a statistically significant association with the level of FCS of the households.

The prevalence of acceptable FCS in this study (68.3%) was considerably lower than the previous WFP national survey in Ethiopia, which reported a 74% prevalence of acceptable FCS (6). This discrepancy may be due to seasonal variations in the study period. Seasonal changes can significantly affect food availability, dietary diversity, and overall nutritional intake, particularly in agricultural communities where local food sources depend on harvest cycles. For example, research indicates that households often experience increased access to a variety of fresh foods during the harvest season, whereas lean seasons may lead to a reliance on stored foods, resulting in decreased dietary diversity and potential nutritional deficiencies (29, 30). Moreover, seasonal factors such as climate conditions also play crucial roles in shaping food consumption. For instance, extreme weather events can lead to crop failures, exacerbating food shortages and influencing purchasing decisions (7).

The prevalence of acceptable FCS is also much lower than the previous facility-based study in Ethiopia which reported an acceptable FCS of 81.5% (14). This difference could be attributed to the fact that pregnant women attending antenatal care services have a higher opportunity to obtain nutrition information than the general population of the community because of the easy accessibility of the settings. Thus, pregnant women might have received awareness about proper food utilization and consumption during antenatal care visits. It could also be attributed to the difference in the study setting. The reported acceptable FCS in the present study is also much lower compared to the recommended WFP target, which is 90% (6).

This study reported a much higher prevalence of acceptable FCS compared to the previous nationwide survey report for the Southern Nation, Nationalities, and Peoples Region, which was 37% (6). This great difference could be due to the time variation of the study periods and the increment of nutrition information sharing, which resulted in improved food consumption practices from time to time (31, 32). The prevalence of acceptable FCS in the current study was also higher than the prevalence of 54.46% that was reported by a study conducted among pregnant women in Haramaya district (54.46%) (15). The difference might be due to the difference in the study population and study settings. All study participants in Haramaya district were from rural settings and were pregnant women (15).

This study has reported that younger-headed households had a significantly lower FCS compared to their counterparts. This finding was consistent with the study assessing the food security status in the Mareko District of Guraghe Zone (33). This finding supports the assumption that when household heads get older, it is expected that they will have a more stable economy, gain money, develop wealth, and have an acceptable FCS than household heads who are younger. However, the current finding was inconsistent with other findings that demonstrated the age of household head has a negative association with household food security status (34, 35).

In this study, it was observed that married household heads were more likely to have acceptable FCS of households, which was consistent with the finding from other studies (35). This is possibly related to an opportunity to pool resources from different sources for household consumption among the married heads. Similarly, households where the household heads were farmers in their occupation had a higher FCS compared to other occupation categories. This finding is in line with a study conducted in eastern Ethiopia where those who owned agricultural land had higher food consumption scores (36). The possible explanation could be that farmers own agricultural land and consume foods they have produced on that land.

The current study reported that households with household heads who attended formal education had a higher FCS compared to their counterparts. This finding is consistent with another study conducted in northwest Ethiopia (37). This could be explained by the fact that their better educational status might increase their chance of having an improved awareness of their food consumption. Better-educated people might have a better income, which could enhance the food supply of the household.

### Strength and limitations

The strength of this study was that it was conducting at the community level. Social desirability bias, recall bias, and the seasonality of the problem are the limitations of this study. Social desirability bias and recall bias were introduced because of culturally sensitive questions in the questionnaire and the longer recall period of the study, respectively. Also, the cross-sectional nature of the study design does not reveal the causal-factor relationship between outcome and associated variables.

In this study, the 7-day dietary record used to evaluate the food consumption. The 7-day dietary record provides only a snapshot of dietary intake, which may overlook the broader temporal dynamics of habitual eating patterns. Therefore, future research should consider longitudinal dietary assessments or multiple recordings across different seasons to capture the full variability in food consumption and its implications for food security.

## Conclusion

The level of acceptable FCS among households in the Konso Zone was found to be low. The age of household head, occupation of household head, marital status of household head, and educational status of household head were found to have statistically significant associations with the acceptable FCS of households. Nutrition education regarding food consumption should target younger-headed households, and households with unmarried household heads. Additionally, policies should support access to agricultural resources for all farmers, regardless of land ownership. Develop and implement educational initiatives focused on nutrition and food security for household heads, particularly targeting those with lower educational levels. Further longitudinal studies should be conducted to identify the variability in FCS due to seasonal variations.

## Data Availability

The raw data supporting the conclusions of this article will be made available by the authors, without undue reservation.
