# Supplementary material for: Level of food consumption score and associated factors among households in Konso Zone, Southwestern Ethiopia: a community-based cross-sectional study
Source: Front Nutr. 2024 Oct 28;11:1481458. doi: 10.3389/fnut.2024.1481458 (PMC11555601; doi:10.3389/fnut.2024.1481458)
Supplement: Supplementary file 1 [file Table_1.DOCX]

**Questionnaire**

This questionnaire is prepared to gather information on the socio-demographic, socio-economic, and food consumption characteristics of households.

| Note the inclusion criteria   - Does the household live in the area for at least six months? 1. Yes 2. No - Is the respondent 18 years of age or more? 1. Yes 2. No   If “No” to any of the inclusion criteria, stop the data collection.  Circle on the answer option selected | | | | |
| --- | --- | --- | --- | --- |
| **Section I. Sociodemographic characteristics of the respondent** | | | | |
| **S.N** | **Question** | **Response** | | **Skip to** |
| 101 | How old are you? | Age in completed years ______ | |  |
| 102 | What is your current marital status? | 1. Single 2. Married 3. Divorced/separated 4. Widowed | |  |
| 103 | What is your gender? | 1. Male 2. Female | |  |
| 104 | What is your religion? | 1. Protestant 2. Orthodox 3. Muslim 4. Traditional | |  |
| 105 | What is your ethnicity? | 1. Konso 2. Derashe 3. Amhara 4. Oromo 5. Other (specify)___________ | |  |
| 106 | What is your educational status? | 1. No formal education 2. Primary education (1-8) 3. Secondary education (9-12) 4. College and above | |  |
| 107 | What is your occupation? | 1. Government employee 2. Merchant 3. Farmer 4. Housewife 5. Other (specify)__________ | |  |
| **Section II. Socio-economic and demographic characteristics of households and household heads** | | | | |
| 201 | How old are you? | Age in completed years ______ | |  |
| 202 | What is your gender? | 1. Male 2. Female | |  |
| 203 | What is your current marital status? | 1. Single 2. Married 3. Divorced/separated 4. Widowed | |  |
| 204 | What is your religion? | 1. Protestant 2. Orthodox 3. Muslim 4. Traditional | |  |
| 205 | What is your ethnicity? | 1. Konso 2. Derashe 3. Amhara 4. Oromo 5. Other (specify)_________ | |  |
| 206 | What is your educational status? | 1. No formal education 2. Primary education (1-8) 3. Secondary education (9-12) 4. College and above | |  |
| 207 | What is your occupation? | 1. Government employee 2. Merchant 3. Farmer 4. Housewife 5. Other (specify)__________ | |  |
| 208 | Where is the residence of your household? | 1. Urban 2. Rural | |  |
| 209 | What is your family size? | __________ | |  |
| **Section III. Food frequency questionnaire** | | | | |
| I would like to ask you about all the different foods that your household members have consumed in the last 7 days. Could you please tell me how many days in the past week your household has consumed the following food items? | | | | |
| **S.N** | **Food items** | **Food group** | | **Frequency per week** |
| 301 | Rice, wheat, maize, teff, barley, sorghum, millet, pasta, bread and other cereals | Main staples | |  |
|  | Cassava, Potato, sweet potatoes and other tubers |  |  |  |
| 302 | Beans, chickpea, Peas, soya beans and other legumes | Pulses | |  |
| 303 | Moringa leave, Cabbage, onion, tomato and other vegetables | Vegetables | |  |
| 304 | Banana, orange, mango, papaya, avocado and other fruits | Fruits | |  |
| 305 | Beef, goat meat, poultry, pork, eggs and fish | Meat and fish | |  |
| 306 | Milk, yogurt and other diary | Milk | |  |
| 307 | Oils, fats and butter | Oil | |  |
| 308 | Sugar and sugar products | Sugar | |  |
| **Section IV. Wealth index** | | | | |
| 401 | Ownership of the house | 1. Private 2. Rented from individuals 3. Other (Specify)_________ | |  |
| 402 | How many rooms are there in your home? | ____________ in number | |  |
| 403 | What is the main material of the dwelling floor? | 1. Earth / Sand  2. Cement  3. ceramic  4. Bamboo  5. Carpet  6. Others (specify)_________ | |  |
| 404 | What is the main material of the roof? | 1. Corrugated iron sheet  2. Wood  3. Thatch  4. Bamboo  5. Others (specify)_________ | |  |
| 405 | What is the main material of the exterior walls? | 1. Stone with mud 2. Wood with mud 3. Stone with cement 4. Others (specify)_________ | |  |
| 406 | What type of fuel mainly used for household cooking? | 1. Electricity 2. Charcoal 3. Wood 4. Animal dung 5. Others (specify)________ | |  |
| 407 | Where is cooking done in your house usually? | 1. In a separate room used as kitchen  2. Elsewhere in the house  3. In a separate building  4. Other (specify)__________ | |  |
| 408 | How many hectares of agricultural land, including irrigation land, do you have? | ______________ | |  |
| 409 | Annual total agricultural products (includes all items) | ___________Quintals | |  |
| 410 | Does your household have | Yes | No |  |
|  | 1. Electricity? | 1 | 0 |  |
|  | 1. A Radio? | 1 | 0 |  |
|  | 1. A Television? | 1 | 0 |  |
|  | 1. A Non-mobile telephone? | 1 | 0 |  |
|  | 1. A Refrigerator? | 1 | 0 |  |
|  | 1. Table? | 1 | 0 |  |
|  | 1. Chair? | 1 | 0 |  |
|  | 1. A bed with cotton/spring mattress? | 1 | 0 |  |
| 411 | Does any member of your household own | Yes | No |  |
|  | 1. A watch? | 1 | 0 |  |
|  | 1. A mobile phone? | 1 | 0 |  |
|  | 1. A bicycle? | 1 | 0 |  |
|  | 1. A bajaj? | 1 | 0 |  |
|  | 1. Animal drawn cart? | 1 | 0 |  |
|  | 1. Car? | 1 | 0 |  |
| 412 | Does your household own any livestock, herds, other farm animals, or poultry? | 1. Yes 2. No | |  |
| 413 | If Q412 Yes, How many of the following animals do the household have? |  | |  |
|  | 1. Cattle, milk cows, or bulls? | _________in number | |  |
|  | 1. Horses, Donkeys, or mules? | _________in number | |  |
|  | 1. Goats and sheep? | _________in number | |  |
|  | 1. Chickens? | _________in number | |  |

Thank you for your response!
